# Genome-wide scans for signatures of selection in Mangalarga Marchador horses using high-throughput SNP genotyping

**DOI:** 10.1186/s12864-021-08053-8

**Published:** 2021-10-14

**Authors:** Wellington B. Santos, Gustavo P. Schettini, Amanda M. Maiorano, Fernando O. Bussiman, Júlio C. C. Balieiro, Guilherme C. Ferraz, Guilherme L. Pereira, Welder Angelo Baldassini, Otávio R. M. Neto, Henrique N. Oliveira, Rogério A. Curi

**Affiliations:** 1 Department of Animal Science, São Paulo State University (Unesp) - FCAV, Via de Acesso Professor Paulo Donato Castelane, NN, CEP: 14884-900, Jaboticabal, SP Brazil; 2 Department of Animal Science, University of São Paulo (USP) - FZEA, Pirassununga, Brazil; 3 Department of Breeding and Animal Nutrition, São Paulo State University (Unesp) - FMVZ, Botucatu, Brazil

**Keywords:** DMRT3, Equine genotyping array, Gaited horse breeds, iHS, ROH, Tajima’s D

## Abstract

**Background:**

The detection of signatures of selection in genomic regions provides insights into the evolutionary process, enabling discoveries regarding complex phenotypic traits. In this research, we focused on identifying genomic regions affected by different selection pressures, mainly highlighting the recent positive selection, as well as understanding the candidate genes and functional pathways associated with the signatures of selection in the Mangalarga Marchador genome. Besides, we seek to direct the discussion about genes and traits of importance in this breed, especially traits related to the type and quality of gait, temperament, conformation, and locomotor system.

**Results:**

Three different methods were used to search for signals of selection: Tajima’s D (TD), the integrated haplotype score (iHS), and runs of homozygosity (ROH). The samples were composed of males (*n* = 62) and females (*n* = 130) that were initially chosen considering well-defined phenotypes for gait: picada (*n* = 86) and batida (*n* = 106). All horses were genotyped using a 670 k *Axiom® Equine Genotyping Array*​ (Axiom MNEC670). In total, 27, 104 (chosen), and 38 candidate genes were observed within the signatures of selection identified in TD, iHS, and ROH analyses, respectively. The genes are acting in essential biological processes. The enrichment analysis highlighted the following functions: anterior/posterior pattern for the set of genes (*GLI3, HOXC9, HOXC6, HOXC5, HOXC4, HOXC13, HOXC11*, and *HOXC10*); limb morphogenesis, skeletal system, proximal/distal pattern formation, JUN kinase activity (*CCL19* and *MAP3K6*); and muscle stretch response (*MAPK14*). Other candidate genes were associated with energy metabolism, bronchodilator response, NADH regeneration, reproduction, keratinization, and the immunological system.

**Conclusions:**

Our findings revealed evidence of signatures of selection in the MM breed that encompass genes acting on athletic performance, limb development, and energy to muscle activity, with the particular involvement of the HOX family genes. The genome of MM is marked by recent positive selection. However, Tajima’s D and iHS results point also to the presence of balancing selection in specific regions of the genome.

**Supplementary Information:**

The online version contains supplementary material available at 10.1186/s12864-021-08053-8.

## Background

The “batida” and “picada” gait types are the main trait of the Mangalarga Marchador horse (MM), representing the unique natural movement allowed in intermediate speeds [1]. The main difference between batida and picada gaits is how the movement is executed, being the diagonal support more frequent than the triple support in the batida gait. In the picada gait, the lateral and triple supports overlap, providing a softer execution to the movement. This difference in movement characterizes the main phenotypic segregation in MM horses [2, 3], and because of this, gait is prioritized in studies involving this breed.

Andersson et al. [4] described the influence of DMRT3 gene and transcription factors, involved in the coordination of limb movement, in gaitedness across horse breeds. Promerová et al. [5] explained in detail the genetic mechanisms behind gait, including allelic patterns associated with equine locomotion across breeds. The frequency of *DMRT3* allele A (mutant) was almost 100% in gaited horses, so the AA homozygous condition was believed to be associated with gait. However, post-investigations of the allelic patterns have shown that breeds without the gait phenotype could also have the mutant allele (A), as well as gaited horses could have the wild type allele (C) [5]. Although DMRT3 appears to be important for gaits in certain breeds, other genes are certainly involved in the expression of this trait.

Selection signatures studies represent a strategy for elucidating not only the complexity over the artificial/natural selection imposed on gait segregations, but also the complexity present in other economically important traits. It is of major interest to better understand the genetic aspects involved with complex phenotypes for the genetic improvement of MM horses. Investigations about hitchhiking effects on genomic regions and recent adaptive fixations were first conducted by Lewontin & Krakauer [6]. Current studies bring the concept of selection signatures, which are particular patterns of DNA identified in genomic regions with mutation and/or which have been under natural/artificial selection pressures in the population [7–9]. The exploitation of selection signatures aids in identifying regions in the genome under selective pressure that may harbor genes and variants that modulate important phenotypes in horses [10, 11].

Over the past few years, the interest in the detection of selection signatures in horses and other species has resulted in the increased number of publications on this topic, being the selection signatures described as results of domestication and selection processes that aimed to increase herd performance and productivity [12, 13]. There are several approaches to identifying signatures of selection [14–22]. Weigand & Leese [23] gathered several of the approaches in a review study, addressing the particularities of each approach in a non-model species perspective. In this study, we used three different approaches to search for signatures of selection in the genome of MM: Tajima’s D (TD) [20], the integrated haplotype score (iHS) [22], and runs of homozygosity (ROH) [21]. The choice of these three methods was made taking into account the genetic structure of our dataset, as horses of both gait modalities were not assigned into subpopulations. Therefore, we used standard within-population approaches to scan for signatures of selection in the MM breed, especially to detect recent signatures. In addition, a detailed discussion on signatures of selection that overlap with candidate genes and gene pathways previously described in the literature were provided, focusing more on candidates related to traits of importance in this breed, especially those related to the type and quality of gait, temperament, conformation, and locomotor system (muscular and skeletal structure).

## Results

### Genetic structure and linkage disequilibrium (LD)

**Fig. 1:**
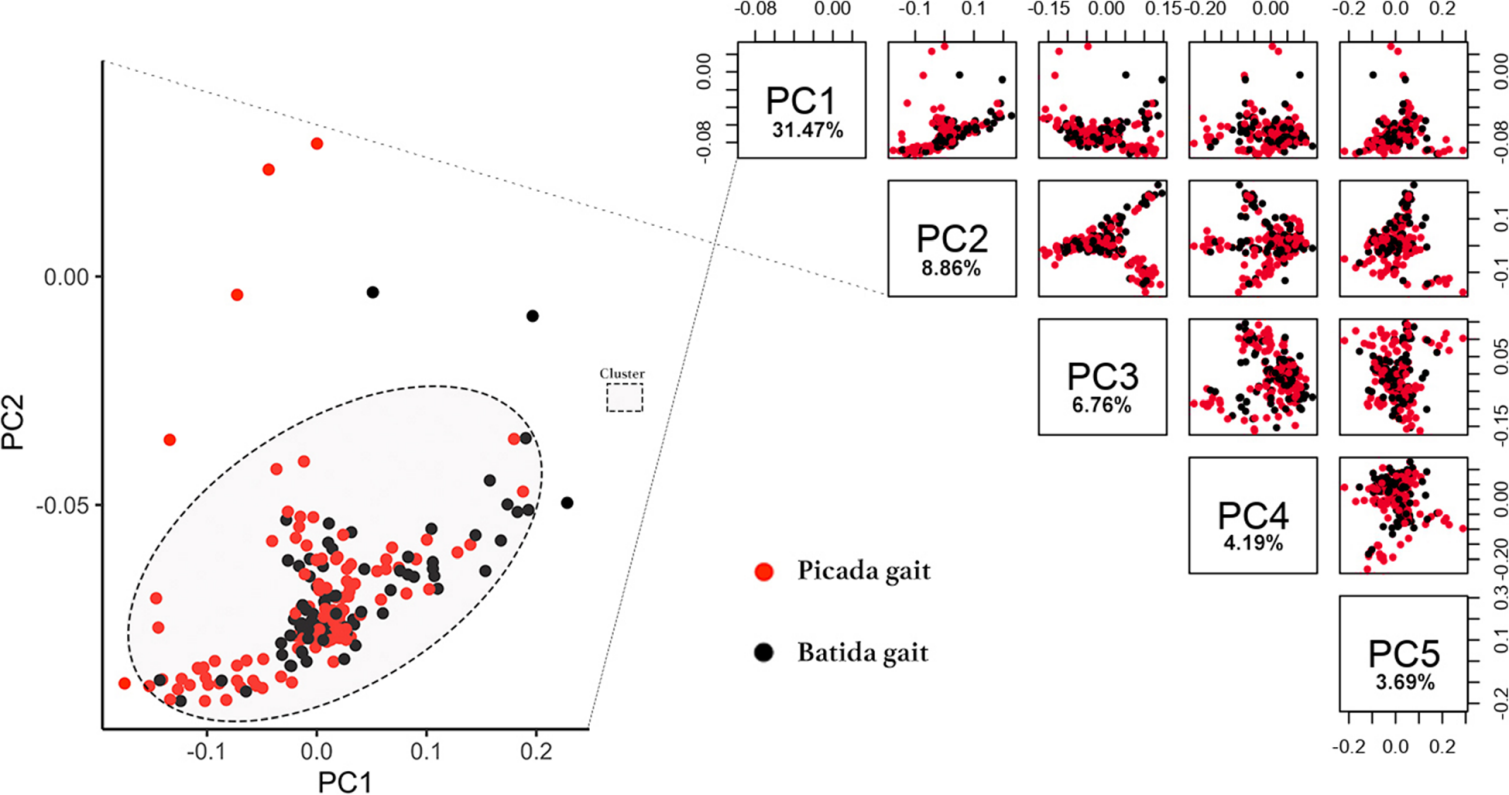
Principal Component Analysis (PCA) based on genotype data for the top five eigenvectors in Mangalarga Marchador horse, considering individuals with the batida and picada gait types. The core PCAs were highlighted in cluster 1

Prior information related to the gait groups of each individual, batida and picada, was considered in the PCA analysis to investigate whether individuals who belong to the same group would cluster together. The top five eigenvectors explained 54.98% of the cumulative variance, with 40.33% assigned to cluster 1 for PCA 1 x PCA 2 (Fig. 1).

Only one cluster persisted in the dataset, meaning that all the individuals are genetically related when genomic information is considered. Some individuals were assigned distant from the center of cluster 1, implying that they are less genetically related to the others. The dispersion of the dataset and segregations (substructures) was attributed to the importance of sires from different families in the breed formation when the most significant number of clusters was assessed. Although animals with different gaits present distinct phenotypes, they are not discriminated by their genotypes which means they are not genetically distant. Therefore, one population, including all animals in the dataset, was taken into consideration for genomic scans of selection signatures.

A decrease in LD was observed as the physical distance between the markers increased. The r^2^ values were below 0.20 at distances below 15 kb (Additional file 1: Fig. S1). Further detailed aspects of the population structure and LD have been reported in Santos et al. [24] using the same database with imputed data. As we chose not to conduct the study with imputed data from two different platforms, the analyses were conducted with only 192 animals genotyped on Axiom MNEC670. Slight changes were noticed between our results and those reported in Santos et al. [24], which were possibly attributed to the different approaches used in the studies, as well as the reduction in the number of animals. However, the conclusions regarding the genetic structure and DL remained the same.

### Signatures of selection and candidate genes identification

**Fig. 2:**
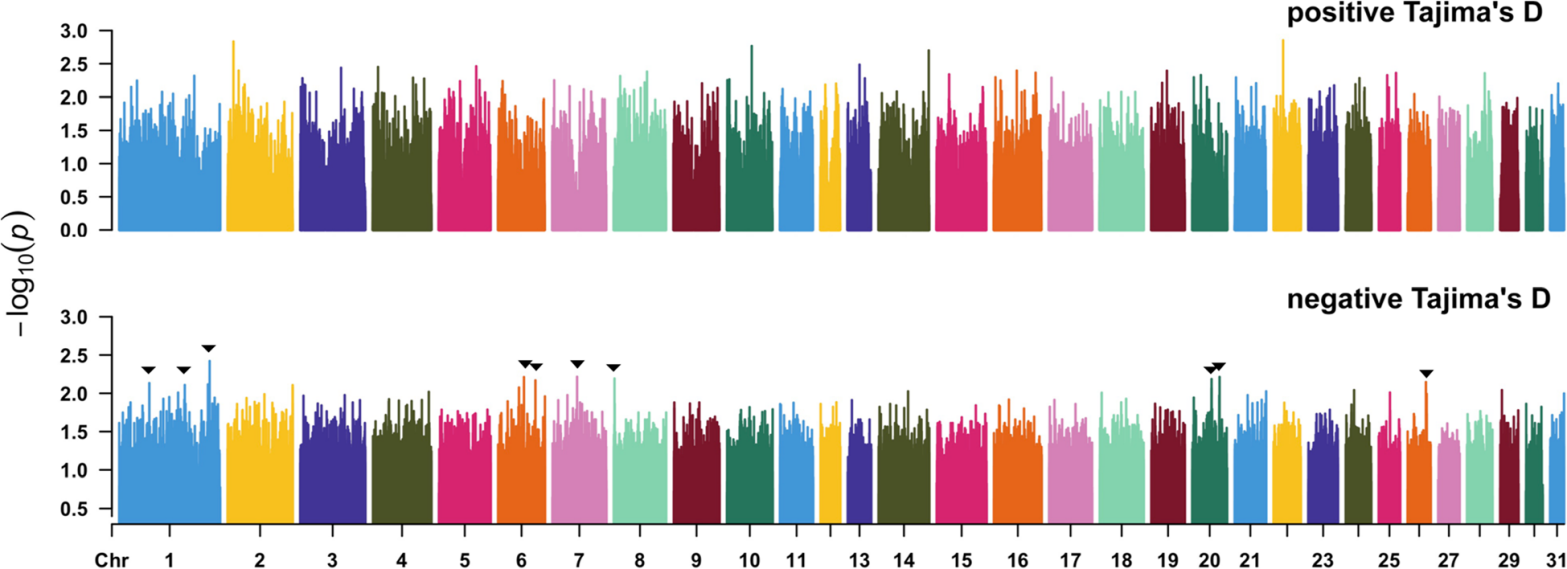
Patterns of genome-wide polymorphisms for Tajima’s D statistics were calculated in 20 kb windows across the genome. The threshold (−log_10_(*p*-value) ≥ 2) was highlighted in dashed line. The negative tail represents signals of recent positive selection. The top ten regions with the most significant values for negative tail were marked with small black arrowheads

High TD values were identified under balanced selection in a wide aspect, and the majority of the equine autosomes demonstrated at least one significant signal of selection (Fig. 2, Additional file 2: Data S1). In general, high proportion of SNPs was noticed under balance selection or sudden population contraction scenarios. Values of -log10(*p*-value) ≥ 2 from empirical *p*-values were considered to be significant signals.

In total, 147 genomic regions with negative and positive tails were identified as significant selection signals in the TD test (*P* < 0.01). As some limitations or biases inherent to the Tajima’s D approach can persist when genotyping data is used, we considered only negative values, which correspond to recent positive selection signals. The recent positive selection signals were observed on the autosomes ECA 1, 6, 7, 8, 20, and 26 (Fig. 2). In total, the TD signals encompassed 27 candidate genes (Table 1). The balancing selection results from this test were not prioritized in our study due to its subjectivity and the limited amount of information necessary for a better understanding.

**Table 1:** Candidate genes identified by Tajima’s D test under evidence of positive signature of selection in the Brazilian Mangalarga Marchador horses

Ensembl Gene ID	Chr	Start Position	End Position	Genes	Description
ENSECAG00000002972	1	168,151,718	168,251,697	*SCFD1*	sec1 family domain containing 1
ENSECAG00000010464	1	168,366,363	168,459,590	*STRN3*	striatin 3
ENSECAG00000021944	1	168,350,423	168,362,177	*COCH*	cochlin
ENSECAG00000001908	6	69,477,881	69,485,306	*KRT84*	keratin 84
ENSECAG00000002542	6	69,388,943	69,394,050	*KRT81*	keratin, type II cuticular Hb1
ENSECAG00000007842	6	69,494,571	69,506,248	*KRT82*	keratin 82
ENSECAG00000008097	6	48,143,741	48,163,481	*CMAS*	cytidine monophosphate N-acetylneuraminic acid synthetase
ENSECAG00000009201	6	69,402,662	69,409,182	*KRT86*	keratin 86
ENSECAG00000009991	6	69,523,789	69,533,483	*KRT75*	keratin 75
ENSECAG00000013512	6	69,553,390	69,558,280	*KRT6C*	keratin 6C
ENSECAG00000015478	6	69,416,432	69,422,664	*KRT83*	keratin 83
ENSECAG00000017378	6	47,951,001	48,065,838	*ABCC9*	ATP binding cassette subfamily C member 9
ENSECAG00000020216	6	69,340,116	69,353,237	*KRT7*	keratin 7
ENSECAG00000006093	8	1,325,765	1,462,223	*CABIN1*	calcineurin binding protein 1
ENSECAG00000017804	8	1,142,374	1,169,168	*UPB1*	beta-ureidopropionase 1
ENSECAG00000020031	8	1,187,365	1,197,777	*GUCD1*	guanylyl cyclase domain containing 1
ENSECAG00000021670	8	1,273,135	1,293,683	*GGT5*	gamma-glutamyltransferase 5
ENSECAG00000023316	8	1,198,613	1,218,433	*SNRPD3*	small nuclear ribonucleoprotein D3 polypeptide
ENSECAG00000023404	8	1,239,052	1,245,534	*LRRC75B*	leucine rich repeat containing 75B
ENSECAG00000025078	8	1,316,427	1,322,900	*SUSD2*	sushi domain containing 2
ENSECAG00000000493	20	35,958,531	36,021,014	*SLC26A8*	solute carrier family 26 member 8
ENSECAG00000012160	20	35,818,020	35,820,026	*CLPS*	*Equus caballus* colipase (CLPS), mRNA
ENSECAG00000014034	20	35,831,559	35,837,234	*LHFPL5*	LHFPL tetraspan subfamily member 5
ENSECAG00000014175	20	36,052,316	36,094,294	*MAPK14*	mitogen-activated protein kinase 14
ENSECAG00000014213	20	35,848,788	35,881,283	*SRPK1*	SRSF protein kinase 1
ENSECAG00000014228	20	50,724,469	50,742,569	*GCM1*	glial cells missing homolog 1
ENSECAG00000014755	20	50,814,846	50,837,355	*ELOVL5*	ELOVL fatty acid elongase 5
Chr: Chromosomes					

**Fig. 3:**
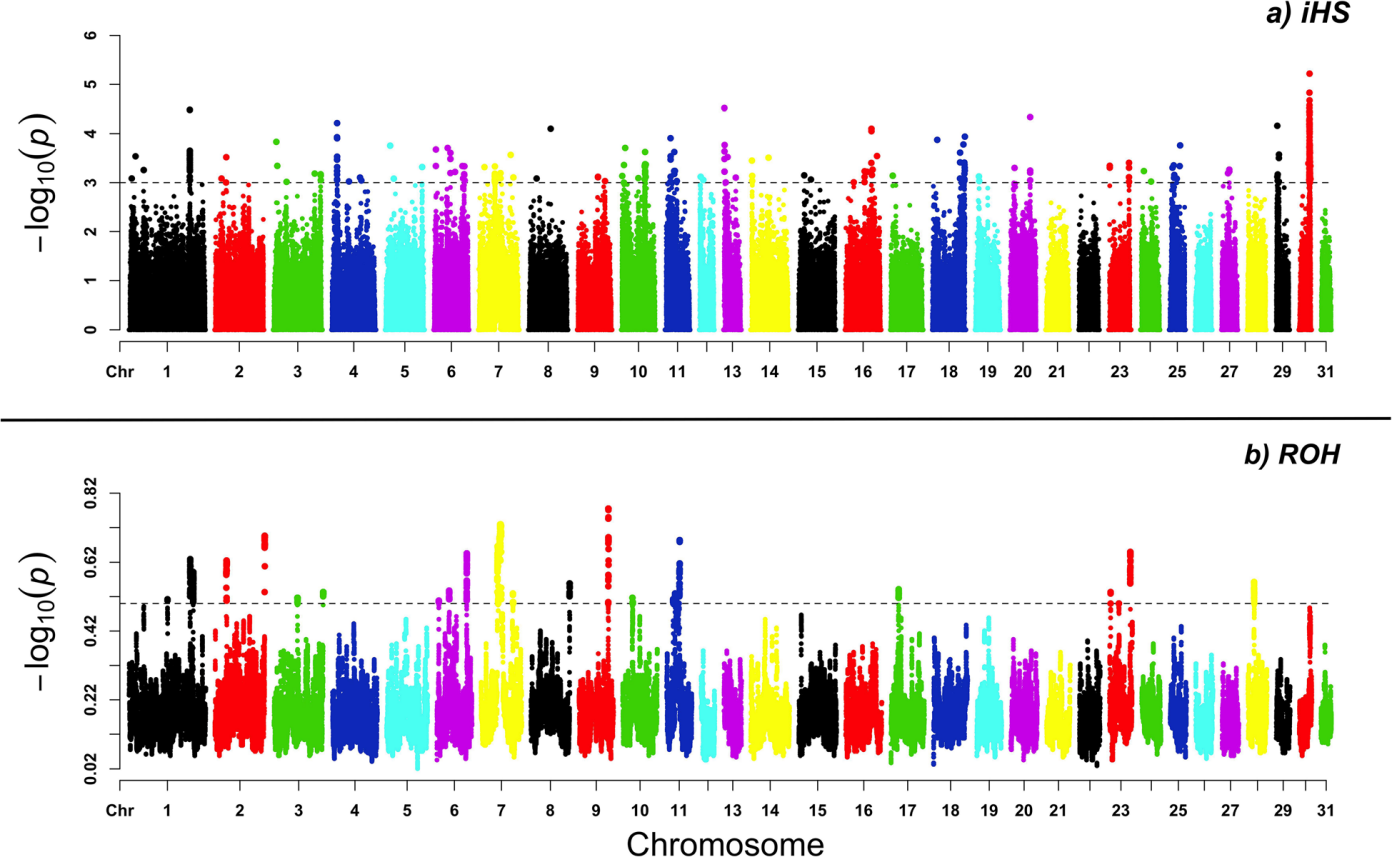
Genome-wide distribution of selection signatures in the equine autosomal chromosomes. a) -log10(*p*-value) for the Integrated Haplotype Score (iHS) plotted against chromosome position, with the significant threshold highlighted with the dashed line (*P* < 0.001). b) Runs of Homozygosity (ROH) islands with dashed line represented by the significant ROH hotspot mean frequency threshold ≥0.50

The iHS positive and negative values were considered in our study, capturing ancient and recent signatures of selection. In total, 292 genomic regions were observed as signatures of selection in the iHS test (Additional file 2: Data S2). They were distributed along the genome, except for chromosomes ECA 21, 22, 26, 28, and 31 (Fig. 3a).

In total, 251 genomic regions were consistent in the iHS positive tail, representing the ancestral allele state, while 41 regions were consistent in the negative tail, representing the derived allele state. Genomic annotations were verified for the significant iHS signals. In total, 332 candidate genes were found within the signatures of selection (Additional file 3: Table S1).

Due to the large number of significant signals found in the iHS test, we did not follow the commonly used method of choosing to display only the top regions. We consider three parameters to prioritize candidate genes in our list: (I) genes within highlighted genomic regions based on the extremes iHS and piHS values, (II) genes related to locomotion, athletic performance, growth, fertility, conformation, pigmentation, and metabolism, and (III) genes that were also found in the Tajima’s D and ROH approaches. Considering these criteria, the shortened list comprised of 104 chosen genes. The genomic regions and their respective genes were shown in Table 2.

**Table 2:** Candidate genes identified by integrated haplotype score (iHS) test under the evidence of signature of selection in the Brazilian Mangalarga Marchador horses

Ensembl Gene ID	Chr	Start Position	End Position	Genes	Description
ENSECAG00000008623	1	149,907,955	150,022,286	*SPRED1*	sprouty related EVH1 domain containing 1
ENSECAG00000010114	1	149,706,774	149,775,059	*RASGRP1*	RAS guanyl releasing protein 1
ENSECAG00000005510	2	28,397,066	28,398,058	*GPR3*	G protein-coupled receptor 3
ENSECAG00000010268	2	28,323,886	28,385,023	*WASF2*	WAS protein family member 2
ENSECAG00000011296	2	28,562,401	28,609,929	*SLC9A1*	solute carrier family 9 member A1
ENSECAG00000014444	2	28,406,073	28,409,277	*CD164L2*	CD164 molecule like 2
ENSECAG00000014857	2	28,412,906	28,416,935	*FCN3*	ficolin 3
ENSECAG00000015410	2	28,420,416	28,429,784	*MAP3K6*	mitogen-activated protein kinase kinase kinase 6
ENSECAG00000020672	2	28,430,246	28,438,417	*SYTL1*	synaptotagmin like 1
ENSECAG00000023706	2	28,443,577	28,453,634	*TMEM222*	transmembrane protein 222
ENSECAG00000024411	2	28,463,993	28,508,594	*WDTC1*	WD and tetratricopeptide repeats 1
ENSECAG00000009649	3	7,693,420	7,744,860	*LPCAT2*	lysophosphatidylcholine acyltransferase 2
ENSECAG00000011520	3	7,794,621	7,835,751	*SLC6A2*	solute carrier family 6 member 2
ENSECAG00000009281	4	13,120,953	13,294,999	*GLI3*	GLI family zinc finger 3
ENSECAG00000007481	5	12,015,652	12,304,265	*ASTN1*	astrotactin 1
ENSECAG00000024570	5	12,310,453	12,412,709	*BRINP2*	BMP/retinoic acid inducible neural specific 2
ENSECAG00000025428	5	12,172,407	12,172,489		eca-mir-488
ENSECAG00000000386	6	34,369,455	34,374,801	*LRRC23*	leucine rich repeat containing 23
ENSECAG00000000465	6	34,410,281	34,420,057	*PTPN6*	protein tyrosine phosphatase, non-receptor type 6
ENSECAG00000000701	6	5,486,218	5,551,290	*FN1*	fibronectin 1
ENSECAG00000000726	6	70,865,117	70,867,507	*HOXC9*	homeobox C9
ENSECAG00000003682	6	70,892,992	70,894,488	*HOXC6*	homeobox C6
ENSECAG00000004151	6	70,897,601	70,899,132	*HOXC5*	homeobox C5
ENSECAG00000004202	6	70,917,898	70,919,290	*HOXC4*	homeobox C4
ENSECAG00000007386	6	34,377,361	34,383,187	*ENO2*	enolase 2
ENSECAG00000009049	6	34,274,460	34,301,295	*CD4*	CD4 molecule
ENSECAG00000009519	6	34,515,391	34,524,075	*C1S*	complement C1s
ENSECAG00000012522	6	34,321,532	34,326,725	*GNB3*	G protein subunit beta 3
ENSECAG00000014517	6	34,328,207	34,330,205	*CDCA3*	cell division cycle associated 3
ENSECAG00000014532	6	34,331,414	34,344,976	*USP5*	ubiquitin specific peptidase 5
ENSECAG00000014653	6	5,446,142	5,472,875	*ATIC*	5-aminoimidazole-4-carboxamide ribonucleotide formyltransferase/IMP cyclohydrolase
ENSECAG00000015581	6	34,346,419	34,349,728	*TPI1*	triosephosphate isomerase 1
ENSECAG00000016937	6	34,425,844	34,429,448	*PHB2*	prohibitin 2
ENSECAG00000019250	6	34,304,988	34,308,833	*GPR162*	G protein-coupled receptor 162
ENSECAG00000021403	6	34,393,931	34,400,776	*ATN1*	atrophin 1
ENSECAG00000021815	6	34,310,310	34,319,714	*P3H3*	prolyl 3-hydroxylase 3
ENSECAG00000022412	6	34,429,726	34,434,811	*EMG1*	EMG1, N1-specific pseudouridine methyltransferase
ENSECAG00000023202	6	34,435,377	34,471,395	*LPCAT3*	lysophosphatidylcholine acyltransferase 3
ENSECAG00000024867	6	70,802,998	70,809,716	*HOXC13*	homeobox C13
ENSECAG00000024869	6	34,402,198	34,404,001	*C6H12orf57*	chromosome 6 C12orf57 homolog
ENSECAG00000024893	6	70,819,239	70,820,860	*HOXC12*	homeobox C12
ENSECAG00000024900	6	70,837,383	70,840,203	*HOXC11*	homeobox C11
ENSECAG00000024985	6	70,850,147	70,854,018	*HOXC10*	homeobox C10
ENSECAG00000025389	6	34,423,082	34,423,146		eca-mir-200c
ENSECAG00000025607	6	70,898,503	70,898,599		eca-mir-615
ENSECAG00000026310	6	34,423,490	34,423,561		eca-mir-141
ENSECAG00000027042	6	34,402,169	34,402,230		U7 small nuclear RNA
ENSECAG00000027594	6	34,426,452	34,426,715		small nucleolar RNA U89
ENSECAG00000003757	10	6,624,595	6,634,234	*GAPDHS*	glyceraldehyde-3-phosphate dehydrogenase, spermatogenic
ENSECAG00000005226	10	6,561,153	6,562,124	*FFAR2*	free fatty acid receptor 2
ENSECAG00000011198	10	60,335,470	60,340,309	*AMD1*	adenosylmethionine decarboxylase 1
ENSECAG00000011975	10	6,634,647	6,636,230	*TMEM147*	transmembrane protein 147
ENSECAG00000012822	10	9,635,035	9,645,344	*EIF3K*	eukaryotic translation initiation factor 3 subunit K
ENSECAG00000013121	10	6,639,494	6,652,250	*ATP4A*	ATPase H+/K+ transporting subunit alpha
ENSECAG00000014214	10	60,375,200	60,382,281	*GTF3C6*	general transcription factor IIIC subunit 6
ENSECAG00000015344	10	9,510,873	9,616,030	*RYR1*	ryanodine receptor 1
ENSECAG00000017061	10	9,616,257	9,633,779	*MAP4K1*	mitogen-activated protein kinase kinase kinase kinase 1
ENSECAG00000017121	10	60,395,300	60,425,982	*RPF2*	ribosome production factor 2 homolog
ENSECAG00000020313	10	60,557,764	60,601,940	*SLC16A10*	solute carrier family 16 member 10
ENSECAG00000021777	10	9,692,742	9,718,476	*ACTN4*	actinin alpha 4
ENSECAG00000025001	10	6,589,049	6,591,925	*KRTDAP*	keratinocyte differentiation associated protein
ENSECAG00000006771	11	13,417,359	13,812,648	*PRKCA*	protein kinase C alpha
ENSECAG00000007214	11	13,765,651	14,005,312	*CACNG4*	calcium voltage-gated channel auxiliary subunit gamma 4
ENSECAG00000000176	13	1,935,848	1,947,933	*ZDHHC4*	zinc finger DHHC-type containing 4
ENSECAG00000008056	13	2,414,177	2,422,727	*FSCN1*	fascin actin-bundling protein 1
ENSECAG00000009724	13	2,153,427	2,160,374	*RBAK*	RB associated KRAB zinc finger
ENSECAG00000010225	13	1,882,012	1,913,837	*GRID2IP*	Grid2 interacting protein
ENSECAG00000011713	13	1,949,573	1,958,047	*C7orf26*	chromosome 7 open reading frame 26
ENSECAG00000013171	13	2,265,413	2,398,956	*RNF216*	ring finger protein 216
ENSECAG00000015935	13	2,463,585	2,465,463	*ACTB*	*Equus caballus* actin beta (ACTB), mRNA
ENSECAG00000016420	13	2,086,916	2,092,792	*ZNF12*	zinc finger protein 12
ENSECAG00000018678	13	2,472,540	2,510,292	*FBXL18*	F-box and leucine rich repeat protein 18
ENSECAG00000022114	13	2,711,477	2,738,292	*WIPI2*	WD repeat domain, phosphoinositide interacting 2
ENSECAG00000013897	16	65,160,454	65,270,909	*RFTN1*	raftlin, lipid raft linker 1
ENSECAG00000008768	18	79,106,315	80,034,010	*PARD3B*	par-3 family cell polarity regulator beta
ENSECAG00000012151	18	12,086,034	12,116,622	*MARCO*	macrophage receptor with collagenous structure
ENSECAG00000016824	18	80,076,435	80,186,347	*NRP2*	neuropilin 2
ENSECAG00000018298	18	76,437,419	76,456,956	*STRADB*	STE20-related kinase adaptor beta
ENSECAG00000019645	18	76,634,162	76,650,235	*TMEM237*	transmembrane protein 237
ENSECAG00000022800	18	76,653,422	76,689,588	*MPP4*	membrane palmitoylated protein 4
ENSECAG00000010916	20	50,162,197	50,233,536	*TRAM2*	translocation associated membrane protein 2
ENSECAG00000015579	20	50,310,519	50,323,621	*TMEM14A*	transmembrane protein 14A
ENSECAG00000016221	20	50,347,190	50,357,534	*GSTA1*	*Equus caballus* glutathione S-transferase alpha 1 (GSTA1), mRNA
ENSECAG00000019567	20	50,425,513	50,435,370	*LOC100271875*	glutathionine S-transferase alpha 3
ENSECAG00000004463	23	50,231,564	50,255,138	*UBAP1*	ubiquitin associated protein 1
ENSECAG00000004776	23	50,338,512	50,340,656	*MYORG*	myogenesis regulating glycosidase (putative)
ENSECAG00000004839	23	50,465,243	50,465,473	*ENHO*	*Equus caballus* energy homeostasis associated (ENHO), mRNA
ENSECAG00000006176	23	50,484,877	50,502,709	*CNTFR*	ciliary neurotrophic factor receptor
ENSECAG00000010758	23	50,257,759	50,299,304	*KIF24*	kinesin family member 24
ENSECAG00000011552	23	50,328,495	50,331,173	*NUDT2*	nudix hydrolase 2
ENSECAG00000011566	23	50,345,688	50,359,034	*C9orf24*	chromosome 9 open reading frame 24
ENSECAG00000012578	23	50,362,111	50,367,137	*FAM219A*	family with sequence similarity 219 member A
ENSECAG00000016961	23	50,426,532	50,464,571	*DNAI1*	dynein axonemal intermediate chain 1
ENSECAG00000027205	23	50,423,793	50,424,056		RNA, 7SK small nuclear pseudogene 24
ENSECAG00000002357	23	50,540,041	50,540,532	*RPP25L*	ribonuclease P/MRP subunit p25 like
ENSECAG00000013178	23	50,543,087	50,549,476	*DCTN3*	dynactin subunit 3
ENSECAG00000019783	23	50,562,602	50,564,385	*SIGMAR1*	sigma non-opioid intracellular receptor 1
ENSECAG00000001054	25	27,004,948	27,005,868	*LOC100071212*	olfactory receptor 1 L6-like
ENSECAG00000001330	25	27,025,906	27,026,868	*OR5C1*	olfactory receptor 5C1
ENSECAG00000002169	25	27,033,670	27,034,620	*OR1K1*	olfactory receptor family 1 subfamily K member 1
ENSECAG00000002222	25	27,136,728	27,138,002	*ZBTB6*	zinc finger and BTB domain containing 6
ENSECAG00000006897	25	26,957,307	26,958,330	*LOC100071227*	olfactory receptor 1 L4-like
ENSECAG00000006946	25	26,979,321	26,980,244	*LOC100071218*	olfactory receptor 1 L4-like
ENSECAG00000017397	25	27,143,414	27,153,522	*ZBTB26*	zinc finger and BTB domain containing 26
ENSECAG00000017729	25	27,161,291	27,312,547	*RABGAP1*	RAB GTPase activating protein 1
ENSECAG00000021253	25	26,896,324	27,056,065	*PDCL*	phosducin like
ENSECAG00000022176	25	27,085,189	27,132,323	*RC3H2*	ring finger and CCCH-type domains 2
ENSECAG00000025393	25	27,106,545	27,106,655		small nucleolar RNA SNORD90
ENSECAG00000007192	30	26,241,146	26,299,185	*PTPRC*	protein tyrosine phosphatase, receptor type C
ENSECAG00000023881	30	26,077,245	26,096,063	*ATP6V1G3*	ATPase H+ transporting V1 subunit G3
ENSECAG00000025552	30	26,398,918	26,399,027		eca-mir-181a-2

Chr: Chromosomes

**Fig. 4:**
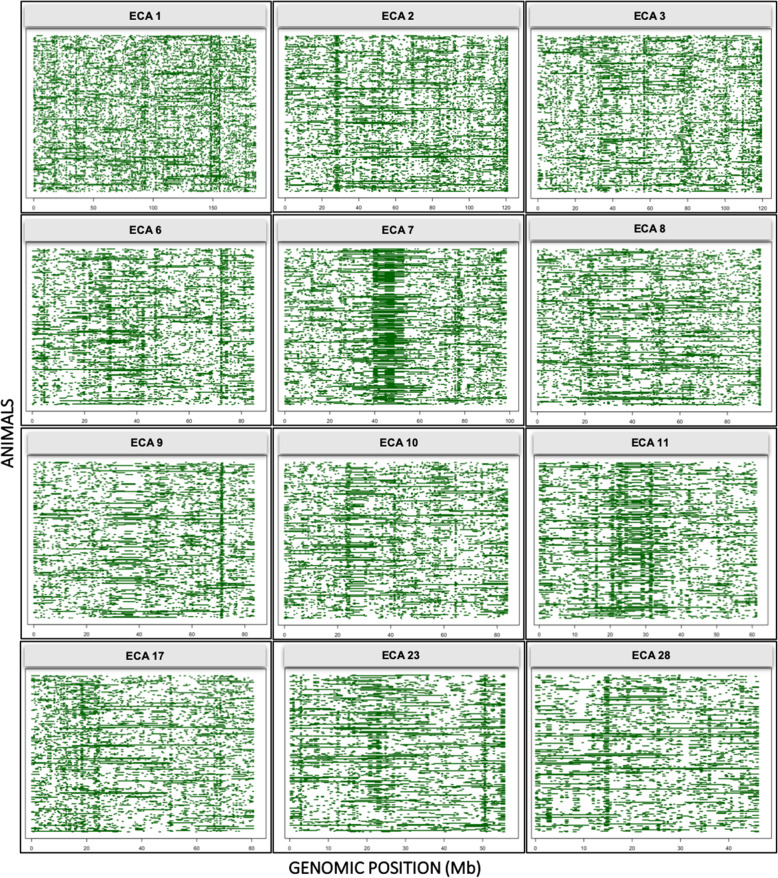
Shared homozygosity interval for the most representative chromosomes in the ROH approach. Green horizontal lines represent the length of ROH. Based on footprints, one can observe regions shared between individuals in the population

In the ROH analysis, 340 SNPs were observed within ROH island regions (mean hotspot) that were regions with frequencies ≥0.5 in the population (Additional file 2: Data S3). In total, 67,478 ROH segments were identified (Fig. 3b). The longest shared homozygous segment was detected in the ECA7, with length above 16 Mb. The number of ROH segments identified in ECA7 was 2846. Most of the ROH segments found in the MM genome corresponded to short segments with lengths around 1–2 Mb (Fig. 4). The ROH size is inversely correlated with age, where longer ROH is originated from recent common ancestors and shorter ROH is originated from distant common ancestors [19, 25, 26].

The same principle of gene annotation used for the iHS approach was used for ROH, adding 125 k upstream and downstream of the significant region. Most of the significant SNPs were located close to each other and, consequently, shared the same windows. Furthermore, windows found in ROH overlapped 38 genes (Table 3).

**Table 3:** Candidate genes identified by runs of homozygosity (ROH) test under evidence of positive signature of selection in the Brazilian Mangalarga Marchador horses

Ensembl Gene ID	Chr	Start position	End Position	Genes	Description
ENSECAG00000010114	1	149,706,774	149,775,059	*RASGRP1*	RAS guanyl releasing protein 1
ENSECAG00000003634	6	30,832,832	30,834,614	*RHNO1*	RAD9-HUS1-RAD1 interacting nuclear orphan 1
ENSECAG00000005303	6	30,883,302	30,896,118	*TULP3*	tubby like protein 3
ENSECAG00000009337	6	31,002,891	31,197,638	*TSPAN9*	tetraspanin 9
ENSECAG00000010144	6	30,609,983	30,638,725	*DDX11*	DEAD/H-box helicase 11
ENSECAG00000010693	6	30,781,746	30,790,420	*ITFG2*	integrin alpha FG-GAP repeat containing 2
ENSECAG00000011303	6	30,931,802	30,968,566	*TEAD4*	TEA domain transcription factor 4
ENSECAG00000013410	6	30,360,399	30,398,526	*SLC6A13*	solute carrier family 6 member 13
ENSECAG00000018082	6	30,792,657	30,799,176	*NRIP2*	nuclear receptor interacting protein 2
ENSECAG00000018777	6	30,810,694	30,816,253	*TEX52*	testis expressed 52
ENSECAG00000019129	6	30,817,902	30,826,537	*FOXM1*	forkhead box M1
ENSECAG00000019283	6	30,595,381	30,608,852	*WASHC1*	WASH complex subunit 1
ENSECAG00000020465	6	30,769,282	30,775,457	*FKBP4*	FK506 binding protein 4
ENSECAG00000005017	7	45,641,390	45,646,041	*FBXW9*	F-box and WD repeat domain containing 9
ENSECAG00000008886	7	45,647,009	45,647,307	*GNG14*	G protein subunit gamma 14
ENSECAG00000009177	7	45,651,702	45,655,097	*DHPS*	deoxyhypusine synthase
ENSECAG00000012154	7	45,617,437	45,620,601	*TRIR*	telomerase RNA component interacting RNase
ENSECAG00000013673	7	45,626,599	45,637,845	*TNPO2*	transportin 2
ENSECAG00000019788	7	45,655,144	45,658,885	*WDR83*	WD repeat domain 83
ENSECAG00000021981	7	45,659,262	45,660,408	*WDR83OS*	WD repeat domain 83 opposite strand
ENSECAG00000003551	9	73,341,857	73,423,501	*LRRC6*	leucine rich repeat containing 6
ENSECAG00000012611	9	73,453,208	73,478,427	*TMEM71*	transmembrane protein 71
ENSECAG00000017467	9	72,950,611	72,999,460	*KCNQ3*	potassium voltage-gated channel subfamily Q member 3
ENSECAG00000002945	11	32,087,533	32,087,985	*CCDC182*	coiled-coil domain containing 182
ENSECAG00000011435	11	31,647,130	32,031,445	*MSI2*	musashi RNA binding protein 2
ENSECAG00000002212	17	18,615,804	18,617,704	*FOXO1*	forkhead box O1
ENSECAG00000003600	17	18,742,806	18,778,545	*MRPS31*	mitochondrial ribosomal protein S31
ENSECAG00000002357	23	50,540,041	50,540,532	*RPP25L*	ribonuclease P/MRP subunit p25 like
ENSECAG00000004839	23	50,465,243	50,465,473	*ENHO*	*Equus caballus* energy homeostasis associated (ENHO), mRNA
ENSECAG00000006176	23	50,484,877	50,502,709	*CNTFR*	ciliary neurotrophic factor receptor
ENSECAG00000008176	23	50,568,433	50,571,634	*GALT*	galactose-1-phosphate uridylyltransferase
ENSECAG00000011566	23	50,345,688	50,359,034	*C9orf24*	chromosome 9 open reading frame 24
ENSECAG00000012578	23	50,362,111	50,367,137	*FAM219A*	family with sequence similarity 219 member A
ENSECAG00000013178	23	50,543,087	50,549,476	*DCTN3*	dynactin subunit 3
ENSECAG00000013412	23	50,605,846	50,607,075	*CCL19*	C-C motif chemokine ligand 19
ENSECAG00000016961	23	50,426,532	50,464,571	*DNAI1*	dynein axonemal intermediate chain 1
ENSECAG00000017442	23	50,576,370	50,582,294	*IL11RA*	interleukin 11 receptor subunit alpha
ENSECAG00000019783	23	50,562,602	50,564,385	*SIGMAR1*	sigma non-opioid intracellular receptor 1
Chr: Chromosomes					

Nine genes were common between ROH and iHS tests. One gene is located on ECA1 (*RASGRP1*), and eight are located within ECA23 (*C9orf24, CNTFR, DCTN3, DNAI1, ENHO, FAM219A, RPP25L*, and *SIGMAR1*). No common genomic regions nor genes were found among TD and other statistics. Therefore, we sought to broaden the understanding of these genes through enrichment analysis and gene networks.

### Enrichment analyses

**Fig. 5:**
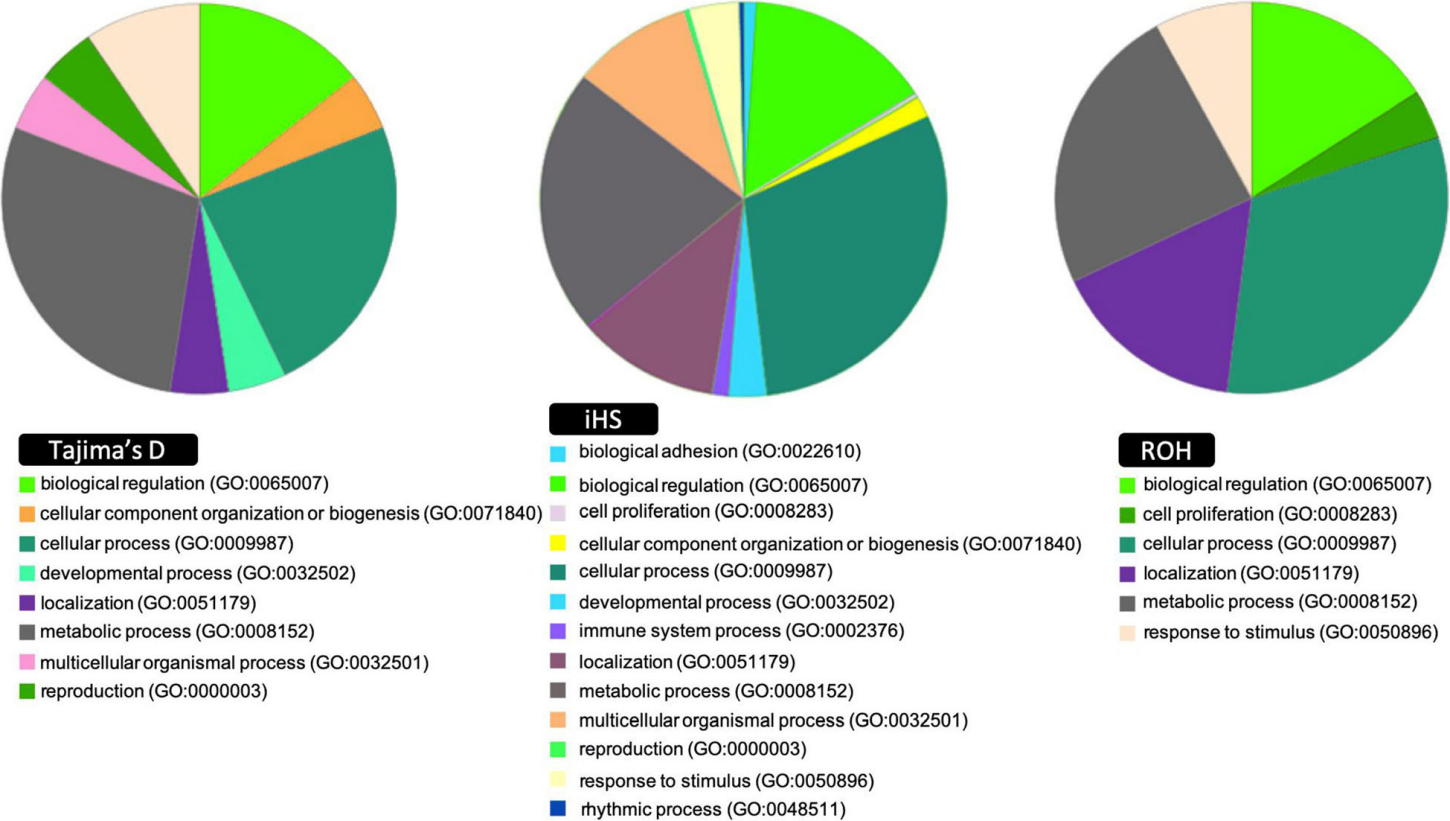
PANTHER GO-Slim pie chart analysis for biological processes for Tajima’s D, integrated haplotype score (iHS), and runs of homozygosity (ROH) approaches used to identify signatures of selection

**Fig. 6:**
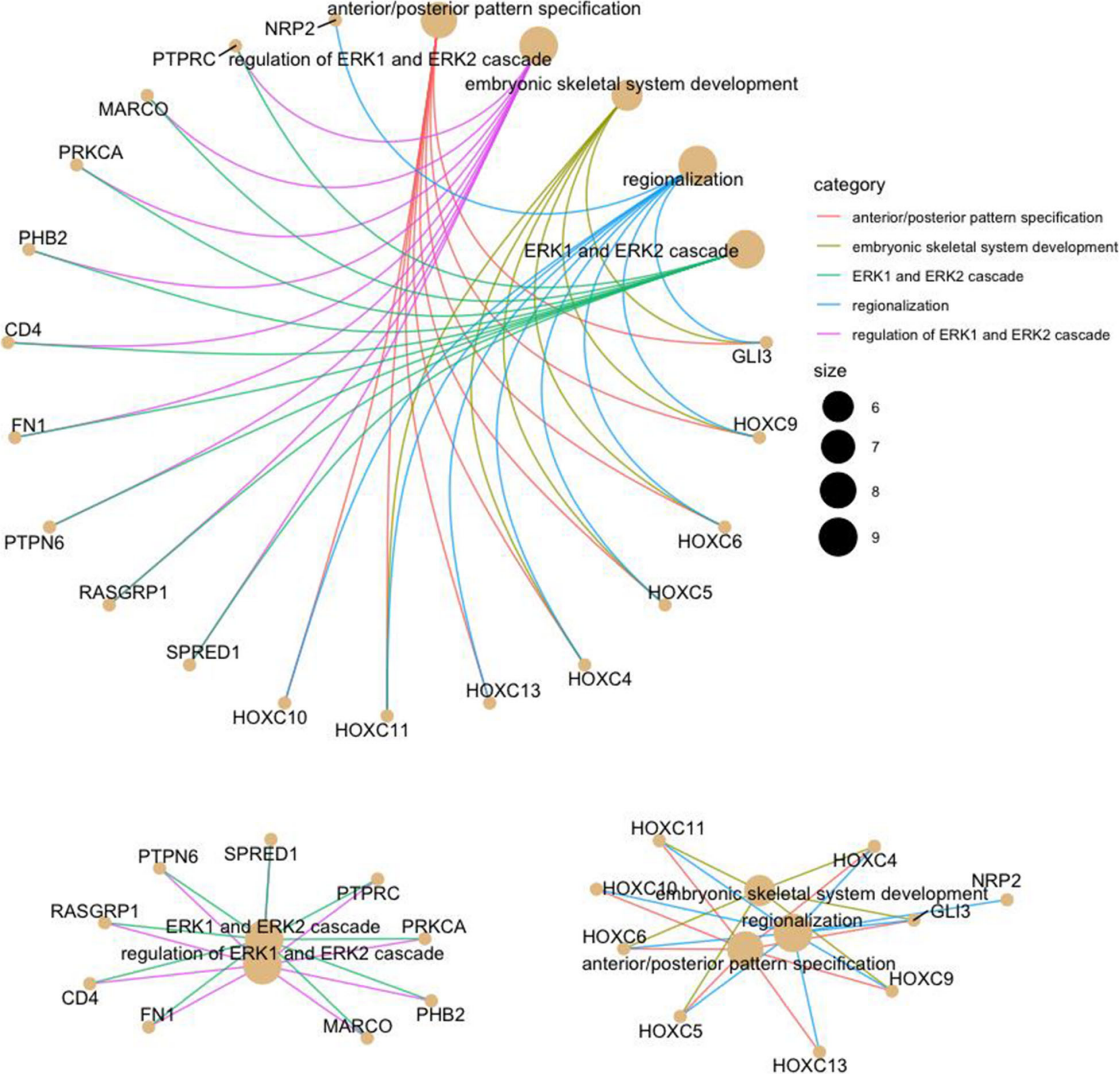
Functional annotation for the top five significant biological functions possibly related to gait and locomotor system (*P* < 0.05)

Genes with biological processes relevant to horses were analyzed for pathways, molecular functions, and cellular components. The enrichment analyses were performed separately considering the gene lists derived from the three approaches, Tajima’s D, iHS, and ROH. To visualize the gene enrichment results, biological processes that are most relevant to the study were assessed (Additional file 3: Table S2). Most of the gene enrichment Gene Ontology (GO) terms for the biological process were attributed to cellular and metabolic processes (Fig. 5). Significant candidate genes with evidence of direct association with gait, locomotor system, energy, exercise, athletic performance, reproduction, and fertility were highlighted in Table 4. More details about the entire pool of identified genes can be accessed in Additional file 3: Table S2. The five main significant biological functions possibly associated with gait and locomotor system were represented in Fig. 6.

**Table 4:** Significant Gene Ontology (GO) terms identified in the enrichment analysis, applying Benjamini–Hochberg correction (*P* < 0.05)

**Locomotor system**	
*GLI3, HOXC9, HOXC6, HOXC5, HOXC4, HOXC13, HOXC11,* and *HOXC10* “anterior/posterior pattern specification” (GO:0009952)	
*GLI3, HOXC13, HOXC11, HOXC10,* and *RC3H* “limb development” (GO:0060173)	
*CCL19* and *MAP3K6* “embryonic limb morphogenesis” (GO:0030326), “embryonic skeletal system development” (GO:0048706), “proximal/distal pattern formation” (GO:0009954), “activation of JUN kinase (JNK) activity” (GO:0007257), “regionalization” (GO:0003002), and “pattern specification process” (GO:0007389).	
**Energy, exercise, and athletic performance**	
*ENO2, TPI1,* and *GAPDHS* “NADH regeneration” (GO:0006735), “canonical glycolysis” (GO:0061621), “glucose catabolic process to pyruvate” (GO:0061718), “glycolytic process through fructose-6-phosphate” (GO:0061615), “glycolytic process through glucose-6-phosphate” (GO:0061620), and “glucose catabolic process” (GO:0006007)	
*MAPK14* “response to muscle stretch” (GO:0035994), “positive regulation of myoblast differentiation” (GO:0045663) and “skeletal system morphogenesis” (GO:0048705);	
*GGT5*, *MAPK14,* and *ELOVL5* “fatty acid metabolic process” (GO:0006631)	
*RYR1* and *MYORG* “skeletal muscle fiber development” (GO:0048741)	
*SLC9A1* and *CD4* “positive regulation of calcium-mediated signaling” (GO:0050850)	
*FOXO1* “regulation of cardiac muscle hypertrophy in response to stress” (GO:1903242)	
*FOXO1* and *CCL19* “response to bronchodilator” (GO:0097366)	
*CCL19* and *WASHC1* “regulation of lipid kinase activity” (GO:0043550)	
The *ELOVL5* “energy production from fatty acids” (GO:1901570, GO:0030497, GO:0042761, GO:1901568, GO:0035338, GO:0045723, GO:0035336, GO:0000038, GO:0046949, GO:0045923).	
*COCH* “bone and cartilage morphogenesis” (GO: 0003433, GO: 0003429)	
*COCH* and *MAPK14* “skeletal system morphogenesis” (GO: 0048705)	
**Reproduction and fertility**	
*SLC26A8* “sperm training” (GO: 0048240)	
*LRRC6* and *DNAI1* “sperm motility” (GO: 0003341, GO: 0097722, GO: 0030317), and others functions associated with the immune system (GO: 0001771, GO: 0002313, GO: 0002827, GO: 0002285, GO: 0002825).

## Discussion

To the best of our knowledge, this is the first study to provide a whole scan for signatures of selection in the MM genome. Our findings shed light on the possible candidate genes/gene groups involved in the regions undergoing selection in this breed. The results and discussion found here can be useful for the comprehension of signatures of selection in other equine breeds.

Overall, quadrupeds use many footfall patterns during locomotion. The gaits are generally considered to be discrete patterns of footfalls and are divided into symmetrical and asymmetrical [27]. The allele A of the *DMRT3* gene is only related to the picada gait in the MM, with two genotypes AA and CA, while the genotype CC is related to batida gait [28]; however, some studies have shown gait ability (the lateral gait pattern) is under the influence of a set of genes [5, 29]. Other discoveries suggested that alleles related to the type of gait were differently fixed within each gait type [5]. In Icelandic horses, no SNP demonstrated genome-wide significance for DMRT3, implying that the ability to pace goes beyond the presence of a single genetic variant [30]. Considering these facts, there is still a lack of information regarding the genetic architecture behind gait.

Selection in the MM breed is based exclusively on competitions where gaited performance records are evaluated relative to that of competitors, often being an empirical selection. Thus, we presumed that time under strong artificial selection is necessary to identify a possible gait type segregation to well-defined lineages. In this regard, it is essential to understand which genes in the MM population are most relevant to accomplish such goals. According to Arnason et al. [31], the thoroughbred carried out a long history of artificial selection for galloping speed while being ridden by a jockey, and it might be the same for MM. A well-defined breeding scheme can shift the allele frequencies of the desirable phenotype, and well-defined lineages could be achieved by selection. We next focused on exploring the signatures of positive recent selection found in the MM population and understanding the genes and pathways associated with these regions. As no significant region was associated with the *DMRT3* gene in our analyses, we included the *DMRT3* gene in a network analysis to get insights on the interactions between the observed candidate genes and *DMRT3.* The identification of genomic regions modified by positive selection has provided discoveries of adaptive directions in different species. Nowadays, the search for signatures of selection is one of the branches of the theoretical and applied evolutionary studies [7]. This study covered three distinct methods to scan for signatures of selection, which diverge according to their concepts and methodology. This implies that each method captures different patterns of genetic variation in different time scales. Due to the density of the genotyping panel used and the complexity of the biological information, these methods still present pitfalls and cannot exploit the genetic variation present within the entire genome. To overcome this limitation, it is essential that results from multiple methods, i. e., in our case, Tajima’s D, iHS and ROH, are used in a complementary way [32].

Common significant regions were found between iHS and ROH. Eight common candidate genes (*C9orf24, CNTFR*, *DNAI1*, *ENHO*, *DCTN3*, *FAM219A*, *RPP25L*, and *SIGMAR1)* were located on ECA23, and one common gene (*RASGRP1*) was located on ECA1. It is interesting that the genes mentioned above on ECA23 are located ~ 28 Mb away from *DMRT3.* The existence of LD in this region is possible biologically, and determining the exact gene(s) under selection can be challenging. Thus, we performed a network analysis including the *DMRT3* gene, and only one occurrence of low co-expression was found between *DCTN3* and *DMRT3*. Therefore, we excluded the possibility of any significant relationship between the eight candidate genes with DMRT3. However, the limitations of using non-model species may have interfered in our presumptions. Besides that, according to Ma et al. [33] and Ablondi et al. [34], during evolution, a series of unknown demographic events further increased the difficulty in detecting modified genomic regions due to different selective pressures. The use of next-generation sequencing (NGS) technologies can be promising for elucidating the relationships between loci in ECA23 because sequencing offers a higher genome coverage and more precision on the position of causative mutations and selection signatures [35]. Complementing the conclusions regarding common candidate regions observed among the used approaches, only a few common candidate genes were found for iHS and ROH. Each method presents specific features implying that non-overlapping regions of signatures of selection between different methods should be treated as complementary information to better understand the different patterns of variation in the MM genome.

The TD results suggest that the MM population is under strong balancing selection; however, many hitchhiking effects were highlighted in the statistics based on the extended haplotype homozygosity and footprints on homozygous regions. The pronounced balancing status in the studied population supported by the TD results was an interesting consequence, possibly explained by the nonexistence of any breeding program in the breed during the past years.

In a previous study developed by our group with the same database, we investigated the runs of homozygosity and populational inbreeding (F_roh_) in the MM breed [25]. A compressive discussion on the length of ROH segments is given in this previous study, which brings important information to understand the breed age and genetic variability. The MM breed is a relatively old breed compared to most modern breeds having originated in the mid-eighteenth century. The class with the highest percentage of ROH was 0–2 Mbps, with 92.78% of the observations, suggesting the majority of ROH segments was classified as short segments [25].

In the present study, we found only one long ROH segment (> 16 Mbps) located on ECA7 (Fig. 4). The number of short segments was more abundant, possibly due to events of recombination that occurred in the past and caused its reduction [19, 36], or due to the limitation of using genotyping data, thereby overestimating the number of short ROH [37]. Again, sequencing data can add value to ROH studies as it covers more genetic variability [38]. However, one of the strengths of ROH analysis is that long homozygous segments can be reliably identified, even from relatively modest marker densities [38].

Evidence for this long ROH segment on ECA7 has already been described in the literature [34]. We cannot fail to consider that equestrian sports prioritize high performance, therefore, conditioning to a positive selection based on directional selection [34, 39]. Another view suggested that strong bottlenecks occurred in this region during the breed formation. Ablondi et al. [34] found similar results for ECA7 in Swedish Warmblood horses and Exmoor ponies. Thus, we speculate that this candidate region of signature of selection in ECA7 is possibly a consequence of a previous bottleneck and not recent positive selection because of the similarity in the results found in distinct breeds. In this sense, our findings corroborate the argument reported in Ablondi et al. [34] for an intense bottleneck, but pointing to a common moment in the evolutionary process for some breeds.

Four genes (*TRIR*, *TNPO2*, *WDR83,* and *WDR83OS*) were highlighted within this longest shared homozygosity segment located on ECA7. These genes were identified under biological functions for localization (GO:0051179) and metabolic processes (GO:0008152). It has been shown that the *TRIR* gene has a significant role in cellular functions [40]. Other genes, *TNPO2* and *WDR83*, were related to tumor development. One region on ECA1 encompassed the gene *RASGRP1,* which was found in common between ROH and iHS and played a key role in the development of T and B cells [41]. Studies have associated *RASGRP* with disease phenotypes in bovine animals [42, 43] and dogs [44].

In general, the genetic signals for the three statistics were most enriched in ontologies corresponding to “biological regulation,” “metabolic process,” and “cellular process.” In the Panther results for iHS and ROH candidate genes, the ontology “localization” was also very representative. Some highlighted candidate genes were associated with gait and locomotor system aspects, with eight of them regulating anterior/posterior pattern specification (Fig. 5).

The HOX genes encode homeodomain transcription factors in developing many embryonic structures in vertebrates and invertebrates [45]. According to Pineault & Wellik [46], as development progresses, tight spatial and temporal control of gene expression and cellular behavior sculpts the developing embryo, adding specific morphological and functional characteristics that determine the adult animal’s lifestyle and functionality.

The *GLI3* gene was identified under the same HOX gene group to regulate anterior/posterior pattern specification. Exploring this information, we found that *GLI3* is a transcriptional activator and a repressor of the sonic hedgehog pathway and plays a vital role in limb development. *GLI3* has been described in the literature as an embryonic patterning of human limbs and other structures [47]. The relationship between the HOX genes and limb musculoskeletal development has been well described in the literature. Pineault & Wellik [46] suggested that the integration of the musculoskeletal system is regulated in part by HOX function in the stromal connective tissue and plays critical roles in skeletal patterning throughout the axial and appendicular skeleton. Evidence to support these genes as possibly regulating limb formation and other processes associated with the locomotor system was reported by Grilz-Seger et al. [48], who found several GO terms shared by more than one breed when studying a set of European and Near Eastern horse breeds; high significance levels were reached for the GO terms “anterior/posterior pattern specification” (GO:0009952), “embryonic skeletal system morphogenesis” (GO:0048704), and “sequence-specific DNA binding” (GO:0043565), mainly based upon the HOXB-cluster in the breeds Gidran, Lipizzan, Posavina, and Noriker.

Other significant signals in the present study were found for the *CCL19* and *MAP3K6* genes enriched for the activation of JUN kinase (JNK) activity. Exercise stimulates c-Jun NH2 Kinase Activity and c-Jun transcriptional activity in human skeletal muscle, showing that the JNK pathway may serve as a link between contractile activity and transcriptional responses in skeletal muscle [49]. Exercise causes selective changes over gene expression, leading to differentiation in skeletal muscle structure and function, which provides strong evidence that this regulation may be associated with gait type segregation in the skeletal muscle on limbs. The effect of activity during exercise in c-jun mRNA expression is via the phosphorylation of two serine residues through the JNKs in the c-Jun transactivation domain, leading to increased transcriptional activity [49].

It is well known in the modern horse that athletic performance has been the target of selection in recent years for many breeds. Increasingly, a perfect horse is being idealized in countless sporting modalities. Indeed, candidate genes were highlighted under important biological functions related to exercise physiology, energy mechanisms, catabolic processes, morphogenesis (bone, skeletal system, and cartilage), and fertility. However, these genes/functions do not act alone in the MM performance. As observed in the network analysis, gene functions are dependent, with the major part of them being regulated in sets.

The interpretation of the network analysis is that most candidate genes, either core genes or peripheral genes, are interconnected. Any peripheral gene is likely to affect the regulation or function of a hub gene. An explanation for the high interconnection in networks is that networks have structures consisting of distinct modules of connected nodes and frequent long-range connections. Under these conditions, any two nodes in the graph are usually connected by just a few steps [50].

Overall, the application of classical and recent techniques in genomics has successfully permitted the identification of several putative selection signatures in the MM population. Based on our discussions, gait may have a polygenic basis and is influenced by many genetic components. Further exploration would be strengthened by searching for signatures of selection by comparing the MM to a non-gaited breed. This method could then be compared to the regions found within the breed and would clarify whether these signatures are unique to the breed (or the gait) rather than being general signatures of selection in horses or if they could potentially detect new genetic bases of gait in the MM. Among the biological processes, genes of biological interest such as the HOX gene family were enriched in the ontology corresponding to “anterior/posterior pattern specification.” Biological processes related to limb morphogenesis, the skeletal system, proximal/distal pattern formation, JUN kinase activity (*CCL19* and *MAP3K6*), and muscle stretch response (*MAPK14*), among others, were reported. Finally, identifying genes and pathways that drive phenotypes is still a challenge; here, we pinpoint some important genes and gene pathways involved in complex selective processes that could be useful in other studies and for the genetic improvement of this breed.

## Methods

### Sample collection, gait patterns, and DNA extraction

Blood sample were collected from competing horses during the 36th Brazilian National Exhibition of the Mangalarga Marchador breed, and also from horses raised in stud-farms located in the States of São Paulo and Minas Gerais. The dataset was composed of males (*n* = 62) and females (*n* = 130) that were initially chosen considering well-defined phenotypes for gait: picada (*n* = 86) and batida (*n* = 106). Also, animals from unrelated lineages were prioritized, avoiding the inclusion of full-sibs. Jugular blood samples (5 mL) were collected from each animal and mixed with 7.5 mg EDTA. We extracted genomic DNA from each sample using an Illustra Blood Genomic PrepMini Spin Kit (GE Healthcare, USA), according to the manufacturer’s instructions. The DNA was quantified using a Qubit® 3.0 Fluorometer (Invitrogen, USA), and quality assessment of DNA was achieved using the NanoDrop™ Lite Spectrophotometer (NanoDrop Lite, Thermo Scientific, USA), and 0.8% agarose gel electrophoresis. The final dilutions per sample were ~ 10 ng/μL.

### Genotype, quality control, filter and phase genotypes

All horses were genotyped with the 670 k *Axiom® Equine Genotyping Array*​ (Axiom MNEC670). Quality control (QC) evaluations were performed using the Axiom™ Analysis Suite Software, version 4, with the default parameters for diploid organisms. QC was performed at sample level considering the following criteria: Dish QC (DQC) ≥ 0.82, call rate ≥ 97, percent of passing samples ≥95, average call rate for passing samples ≥98.5; and at SNP level using the threshold for call rate ≥ 97, with twenty-six other parameters that can be consulted in more detail (Additional file 4: Methods S1). The coordinates of the genotyping data were remapped to reference assembly of the equine genome EquCab3.0 [51], excluding non-autosomal chromosomes. The raw reports with the EquCab3.0 SNP coordinates for the MNEc670k array, used in our analysis, are available at https://www.animalgenome.org/repository/pub/UMN2018.1003/. Coordinates between the two assemblies was accessed using NCBI (https://www.ncbi.nlm.nih.gov/genome/tools/remap). The final genotyping file contained information from 545,219 SNPs, located within the 32 chromosomes, including chromosome X (Additional file 1: Fig. S2).

Additional QC analyses at SNP level were performed in VCFtools and R software in accordance with each method, being imposed a QC for Hardy-Weinberg <1e-8 for the three signature of selection statistics, minor allele frequency (MAF) < 0.01 for TD and iHS, MAF < 0.005 for ROH. SNPs were excluded based on these thresholds. SNP in the same position were removed. Thereby, two datasets were available due to the different QC applied for MAF; with a total of 422,656 SNP available in the dataset for TD and iHS analyses (MAF < 0.01), and 444,929 SNP available in the dataset used in ROH analysis (MAF < 0.005). We adopted an extreme lower MAF parameter for ROH to follow the recommendations of previous studies, which described possible underestimation problems when MAF is used [52]. Genotype phasing was performed in Beagle v.5.0, which provides faster and accurate algorithms [53], and the phased data was used in the TD and iHS analyses.

### Population structure and linkage disequilibrium analyses

The principal component analysis (PCA) was performed in Plink 1.9 [21] using linkage disequilibrium. A pruning parameter was applied to remove correlations between SNP and keep approximately independent SNPs; the parameter --indep-pairwise was used. The relatedness between individuals was used for the computation of genome-wide IBD estimates. Before computing PCAs in the R software, close related individuals were excluded based on the high-values for pairwise PI_HAT statistic sum.

The linkage disequilibrium (LD) level was calculated for the entire panel using the phased data. To conduct the LD decay analysis, the PopLDdecay pipeline was used with default pruning [54]. The density was reduced to 347,935 SNPs after the LD pruning. Graphs and complementary analyses for the plot were conducted using the R packages pegas [55], ape [56], and ggplot2 [57].

### Genome-wide scan for signals of positive selection

We used three distinct approaches to capture the evolutionary aspects of the selection in the MM. Each approach has some strengths and disadvantages, and the combination and reproducibility of the results add greater accuracy to the analyses.

#### Tajima’s D (TD)

Sliding windows of 20 kb across all autosomal regions were used in the TD analysis. The analysis was performed in the VCFtools (http://vcftools.sourceforge.net/), using the command option “--TajimaD”. Windows containing missing variants were ignored. Windows were sorted in ascending order of the TD values, using empirical *p*-values [58] of less than 0.01, before constructing the graphs.

#### Integrated haplotype score (iHS)

The R package rehh v.3.01 [22, 59] was used in the iHS analysis. Due to the absence of representative studies in horses and most non-model species for the designation of alleles as ‘ancestral’ or ‘derived’, iHS analysis was conducted using unpolarized alleles, which is a new feature of the latest version of the rehh package. This version allows the function to be defined as “FALSE”, which is ideal for the study of domestic animals as well as non-model organisms. The iHH (integrated EHH) values were computed for the major (most frequent) and minor (second-most frequent) alleles. Values of iHS ≥ 2 or ≤ 2 are already considered as significant signals of selection using the default settings [59] because they reflect on a *p*-value < 0.01. However, an ideal value for iHS or piHS (*p*-value for iHS) is not well defined in the literature. We used iHS values ≥3.5 or ≤ 3.5, for which piHS ≥3 was considered statistically significant, rejecting the null hypothesis at a level of significance equal to 0.1% (*p*-value< 0.001). The piHS values are products of iHS transformation to assign a *p*-value, being piHS = [−log10[1–2|ΦiHS-0.5|], wherein Φ iHS is the Gaussian cumulative distribution function of iHS.

#### Runs of homozygosity (ROH)

The analysis was conducted with Plink 2.0, using the following parameters “--homozyg --density 50 --gap 1000 --kb 250 --snp 50, --window-het 2, --window-missing 2, --window-snp 50, --window-threshold 0.05)” [60]. Binary runs of homozygosity were generated with the R script developed by Boison (https://github.com/soloboan/ROHs). SNPs with an ROH proportion lower than 0.01 were discarded. The signatures of selection for ROH, i.e., ROH islands, were defined as ROH regions (mean hotspot) with frequencies ≥0.5 in the population.

### Gene annotation and enrichment analysis

Gene annotation was carried out with the genomic regions identified as signatures of selection, considering the three methods separately. Window sizes were set at 125 kb upstream and downstream of each significant region/SNP. The window size was defined based on LD information and approximate values described in the literature. Genes within these windows were identified based on the most recent assembly of the equine genome sequence (EquCab3.0) using the BioMart R package [61]_._ Enrichment analysis was carried out on the PANTHER Classification System (www.pantherdb.org) to provide an accurate inference of biological processes, molecular functions, and cellular component analysis of the candidate genes. The enrichment analysis results were plotted using the ggplot2 R package [57] for better visualization. The *p*-values were adjusted to Benjamini–Hochberg (BH) (*P* < 0.05), which implements methods to analyze and visualize the functional profiles of genes and gene clusters [62]. We also used network analysis as a complementary approach to study the genes and how they are possibly functionally related (further details were provided in the Additional file 1: Gene network analysis).

## Supplementary Information

**Additional file 1: Fig. S1** Genome-wide linkage disequilibrium (LD) decay plot for 192 Mangalarga Marchador based on 347,935 SNP markers. **Fig. S2** Final density of 545,219 SNP in the Mangalarga Marchador horse genome after Axiom™ Analysis Suite pruning. **Fig. S3** Interaction networks of candidate genes identified from signatures of selection. Different colored arrows indicate the types of evidence used in predicting the associations.

**Additional file 2:** **Data S1** TD output. **Data S2** iHS output. **Data S3** ROH output.

**Additional file 3:** **Table S1** All genomic annotations for iHS. **Table S2** Gene enrichment results for TD, iHS, and ROH.

**Additional file 4:** Axiom^TM^ Analysis Suite final report.

## Data Availability

The data generated during this study are included in this published article and its supplementary information files. The data that support the findings of this study are available on request from the email address: rogerio.curi@unesp.br. The upload of this information was not possible due to privacy or ethical restrictions.
