# Supplementary material for: Genome-wide scans for signatures of selection in Mangalarga Marchador horses using high-throughput SNP genotyping
Source: BMC Genomics. 2021 Oct 14;22:737. doi: 10.1186/s12864-021-08053-8 (PMC8515666; doi:10.1186/s12864-021-08053-8)
Supplement: Supplementary file 1 — Additional file 1: Fig. S1 Genome-wide linkage disequilibrium (LD) decay plot for 192 Mangalarga Marchador based on 347,935 SNP markers. Fig. S2 Final density of 545,219 SNP in the Mangalarga Marchador horse genome after Axiom™ Analysis Suite pruning. Fig. S3 Interaction networks of candidate genes identified from signatures of selection. Different colored arrows indicate the types of evidence used in predicting the associations. [file 12864_2021_8053_MOESM1_ESM.pdf]

Genome-wide scans for signatures of selection in Mangalarga Marchador horses using high-throughput SNP genotyping

Wellington B. Santos[1]*, Gustavo P. Schettini[1], Amanda M. Maiorano[1]; Fernando O. Bussiman[2], Júlio C. C. Balieiro[2], Guilherme C. Ferraz[1], Guilherme L. Pereira[3], Welder Angelo Baldassini[3]; Otávio R. M. Neto[3], Henrique N. Oliveira[1] & Rogério A. Curi[3]

Supplementary material: **Additional file 1**

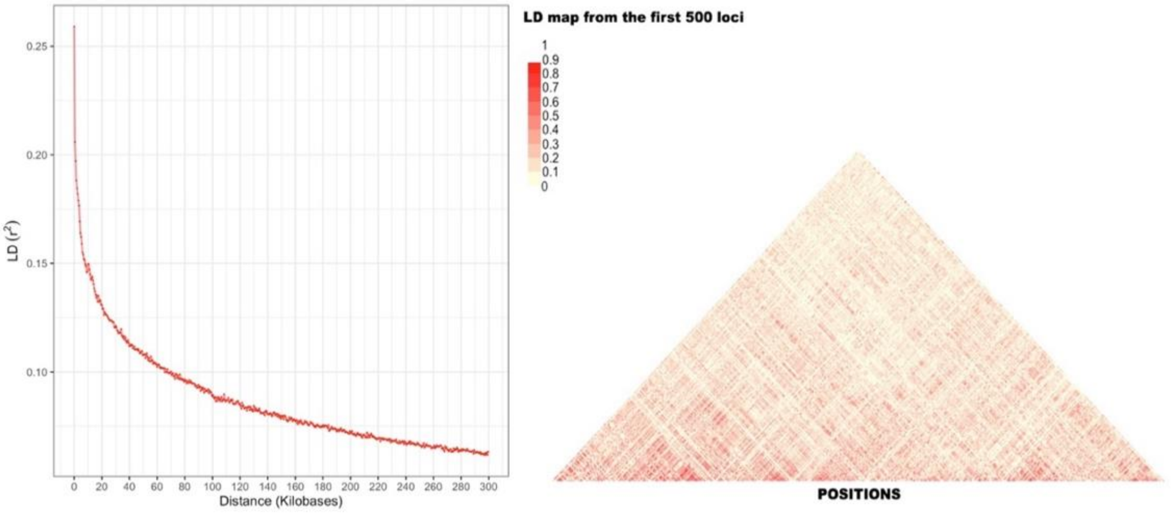

Fig. S1 Genome-wide linkage disequilibrium (LD) decay plot for 192 Mangalarga Marchador based on 347,935 SNP markers

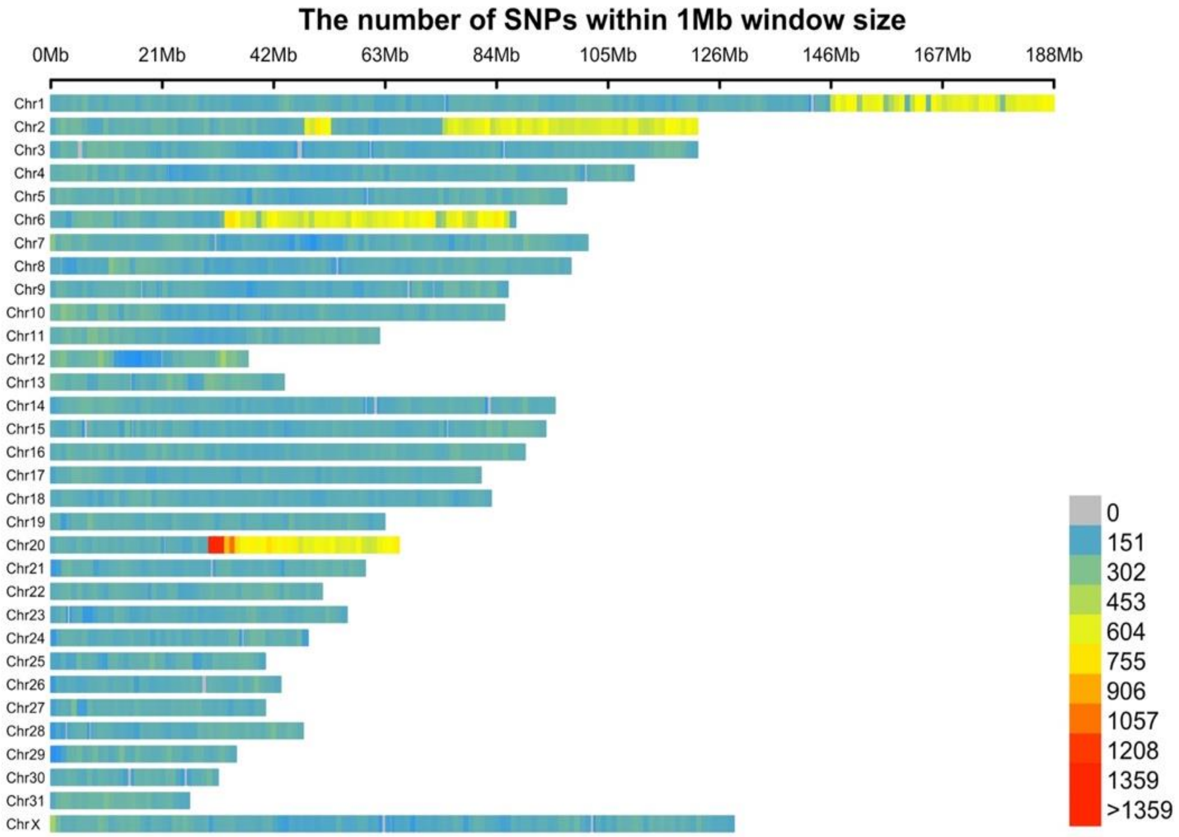

Fig. S2 Final density of 545,219 SNP in the Mangalarga Marchador horse genome after Axiom™ Analysis Suite pruning

Gene network analysis

Gene networks can shed light on the complex behavior of horse genes related to gait patterns, diseases, performance, and physiology. This analysis makes it possible to study functional interactions among proteins encoded by a list of genes. We used this analysis to add meaning to the list of genes related to signatures of selection and to investigate whether these candidate genes have some relationship with the *DMRT3* gene.

The networks were constructed considering candidates genes resulting from the Tajima's D, iHS, and ROH methods, and also by the biological pathways known in the equine sector in a wider sense. Each gene annotated in the previous stage of gene enrichment was used, except for iHS, which, due to the high number of signals, only contemplated the pruned genes according to the three criteria established in the iHS results section. The interactions were calculated by GeneMANIA [1] and STRINGdb R package [2]. Direct (physical) and indirect (functional) associations between genes were included in the interactions [3]. We merged the 169 candidate genes (TD=27, iHS=104, and ROH=38 genes) to conduct the analysis (known and predicted protein-protein interactions). The STRING identifiers could not map 16 genes, 21 did not present any connection, and nine genes were common between the methods, remaining 123 genes for gene network analysis. The gene network and interconnections can be observed in Fig. S3.

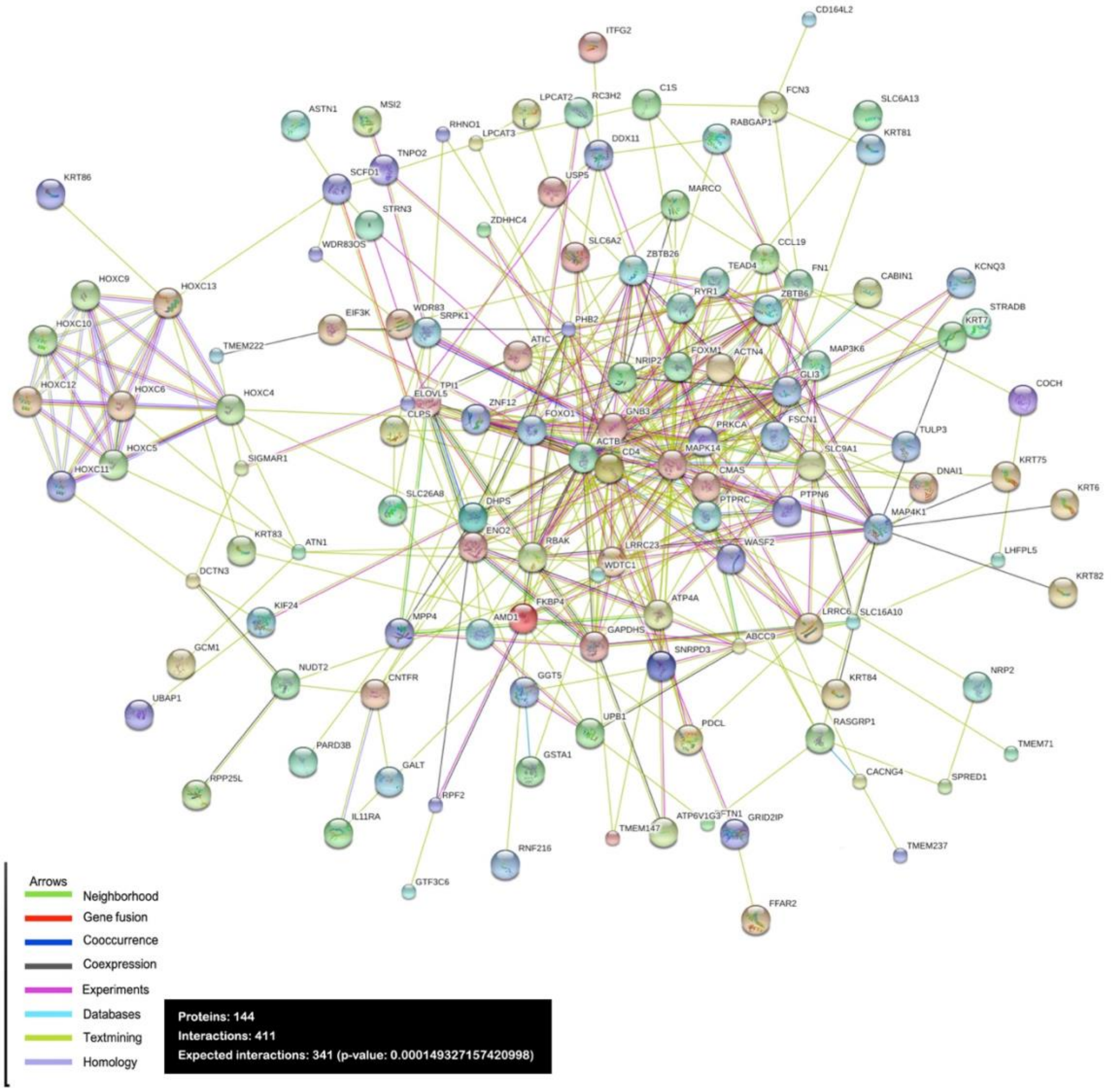

Fig. S3 Interaction networks of candidate genes identified from signatures of selection. Different colored arrows indicate the types of evidence used in predicting the associations.

The image shows the associations are separated into two clusters of genes. Basically, the HOX family genes were involved in the first cluster. The second cluster included the majority of the annotated genes which activate many pathways. On the black box in the plot, a p-value was inserted that represents the probability of the expectation of an equal or greater number of interactions by chance. This p-value is

practically null, meaning that the interactions were not caused by chance. An additional network analysis included the DMRT3 gene in the database, but did not identify clusters around this gene, and was only finding a few associations of low co-expression.

Supplementary References

1. Franz M, Rodriguez H, Lopes C, Zuberi K, Montojo J, Bader GD, Morris Q. GeneMANIA update 2018. Nucleic Acids Res. 2018;46:W60–W64.

2. Franceschini A, Szklarczyk D, Frankild S, Kuhn M, Simonovic M, Roth A, et al. STRING v9.1: Protein-protein interaction networks, with increased coverage and integration. Nucleic Acids Res. 2013;41:808–15.

3. Szklarczyk D, Franceschini A, Wyder S, Forslund K, Heller D, Huerta-Cepas J, et al. STRING v10: protein-protein interaction networks, integrated over the tree of life. Nucleic acids research. 2015;43:D447–D452.
