# Supplementary material for: Genome-wide scans for signatures of selection in Mangalarga Marchador horses using high-throughput SNP genotyping
Source: BMC Genomics. 2021 Oct 14;22:737. doi: 10.1186/s12864-021-08053-8 (PMC8515666; doi:10.1186/s12864-021-08053-8)

Genome-wide scans for signatures of selection in Mangalarga Marchador horses using high-throughput SNP genotyping

Wellington B. Santos[1]*, Gustavo P. Schettini[1], Amanda M. Maiorano[1]; Fernando O. Bussiman[2], Júlio C. C. Balieiro[2], Guilherme C. Ferraz[1], Guilherme L. Pereira[3], Welder Angelo Baldassini[3]; Otávio R. M. Neto[3], Henrique N. Oliveira[1] & Rogério A. Curi[3]

Supplementary material: **Additional file 4**

Analysis Summary

- **Batch Name:** DATA_MM_2019
- **Array Package Name:** Axiom_MNEc670.r3
- **Array Type Name:** Axiom_MNEc670
- **Array Display Name:** Axiom_MNEc670.r3
- **Workflow Type:** Best Practices Workflow
- **Date Created:** 7/14/2019 9:30:45 PM

Sample Summary

- Number of input samples: 192
- Samples passing DQC: 192 out of 192
- Samples passing DQC and QC CR: 192 out of 192
- Samples passing DQC, QC CR and Plate QC: 192 out of 192 (100%)
- Number of failing samples: 0
- Number of input samples without QC information: 0
- Number of Samples Genotyped: 192
- Average QC CR for the passing samples: 99.257
- Gender Calls Counts: female=130 male=62 unknown=0
- Inbred Penalty Applied: no

Plate QC Summary

Plate Barcode	Result	Nº files in a batch	Nº files failing dish QC	Nº files failing QC Call rate	Nº samples that passed	Percent of passing samples	Average call rate for passing samples	Filtered Call Rate
5505834338526080618058	PASSED	96	0	0	96	100	99.343	99.467
5505834338526080618064	PASSED	96	0	0	96	100	99.17	99.278

ProbeSet Metrics Summary

- Number of ProbeSets: 629474

ConversionType	Count	Percentage
PolyHighResolution	354473	56.313
NoMinorHom	128407	20.399
Other	66655	10.589
MonoHighResolution	62339	9.903
CallRateBelowThreshold	15948	2.534
OTV	1652	0.262

Marker Metrics Summary

- Number of Markers: 629474
- Number of BestandRecommended: 545219
- Percent BestandRecommended: 86.615

ConversionType	Count	Percentage
PolyHighResolution	354473	56.313
NoMinorHom	128407	20.399
Other	66655	10.589
MonoHighResolution	62339	9.903
CallRateBelowThreshold	15948	2.534
OTV	1652	0.262

Sample QC Thresholds

- DQC: ≥ 0.82
- QC call_rate: ≥ 97
- Percent of passing samples: ≥ 95
- Average call rate for passing samples: ≥ 98.5

SNP QC Thresholds

- species-type: Diploid
- cr-cutoff: ≥ 95
- fld-cutoff: ≥ 3.6
- het-so-cutoff: ≥ -0.1
- het-so-XChr-cutoff: ≥ -0.1
- het-so-otv-cutoff: ≥ -0.3
- hom-ro-1-cutoff: ≥ 0.6
- hom-ro-2-cutoff: ≥ 0.3
- hom-ro-3-cutoff: ≥ -0.9
- hom-ro: true
- hom-het: true
- num-minor-allele-cutoff: ≥ 2
- hom-ro-hap-1-XChr-cutoff: ≥ 0.1
- hom-ro-hap-1-MTChr-cutoff: ≥ 0.4
- hom-ro-hap-2-XChr-cutoff: ≥ 0.05
- hom-ro-hap-2-MTChr-cutoff: ≥ 0.2
- aaf-XChr-cut: < 0.36
- fld-XChr-cut: ≥ 4
- homfld-XChr-cut: ≥ 6.5
- homfld-YChr-cut: ≥ 6.5
- min-YChr-samples-cut: ≥ 5
- sign-diff-hom-1-cutoff: ≥ 0.5
- sign-diff-hom-2-cutoff: ≥ 0.4
- min-mean-cp2-cutoff: ≥ 9
- max-mean-cp2-cutoff: ≤ 15
- priority-order: PolyHighResolution, NoMinorHom, OTV, MonoHighResolution, CallRateBelowThreshold
- recommended: PolyHighResolution, NoMinorHom, MonoHighResolution, Hemizygous
- y-restrict: ≤ 0.2

Axiom™ Analysis Suite final report

DQC by Plate

DQC: 1 / 0.82 / 0.5 — Configure

550583433852608061805 8

	1	2	3	4	5	6	7	8	9	10	11	12
A	0.995	0.995	0.995	0.995	0.993	0.992	0.995	0.989	0.986	0.993	0.992	0.992
B	0.99	0.995	0.987	0.992	0.99	0.993	0.991	0.995	0.992	0.994	0.997	0.993
C	0.993	0.991	0.994	0.995	0.992	0.987	0.996	0.989	0.995	0.993	0.989	0.994
D	0.99	0.997	0.995	0.99	0.994	0.995	0.995	0.995	0.993	0.994	0.992	0.992
E	0.995	0.993	0.992	0.996	0.995	0.988	0.997	0.991	0.994	0.992	0.995	0.993
F	0.994	0.99	0.993	0.995	0.991	0.995	0.993	0.996	0.992	0.992	0.992	0.99
G	0.992	0.995	0.996	0.99	0.988	0.993	0.991	0.992	0.997	0.997	0.991	0.995
H	0.993	0.997	0.996	0.994	0.994	0.989	0.996	0.992	0.995	0.993	0.996	0.992

5505834338526080618064

	1	2	3	4	5	6	7	8	9	10	11	12
A	0.993	0.972	0.985	0.982	0.99	0.989	0.988	0.989	0.986	0.988	0.99	0.98
B	0.99	0.995	0.986	0.99	0.985	0.995	0.988	0.981	0.991	0.99	0.994	0.993
C	0.992	0.988	0.988	0.99	0.986	0.988	0.99	0.988	0.985	0.994	0.989	0.994
D	0.994	0.99	0.981	0.938	0.992	0.993	0.989	0.989	0.992	0.993	0.981	0.986
E	0.991	0.994	0.99	0.99	0.992	0.988	0.986	0.983	0.99	0.983	0.988	0.992
F	0.994	0.99	0.975	0.991	0.988	0.99	0.992	0.989	0.993	0.987	0.984	0.992
G	0.993	0.963	0.993	0.986	0.993	0.984	0.988	0.989	0.989	0.995	0.988	0.988
H	0.992	0.993	0.991	0.987	0.995	0.994	0.99	0.99	0.992	0.995	0.995	0.991

QC call_rate by Plate

QC call_rate: 100 / 50 — Configure

5505834338526080618058

	1	2	3	4	5	6	7	8	9	10	11	12
A	99.3	99.559	99.71	99.331	99.445	98.922	99.549	99.067	98.916	99.342	99.279	99.005
B	98.958	99.357	98.958	99.342	98.885	99.425	99.082	99.518	99.435	99.43	99.704	99.046
C	99.523	99.238	99.585	99.58	99.103	98.828	99.611	98.885	99.585	99.316	98.932	99.393
D	99.31	99.663	99.704	99.108	99.16	99.601	99.041	99.694	99.399	99.585	99.222	99.575
E	99.471	99.399	99.279	99.466	99.575	99.082	99.71	99.207	99.533	99.228	99.476	99.29
F	99.445	99.098	99.456	99.342	99.062	99.704	99.072	99.699	99.43	99.3	98.937	98.88
G	99.404	99.606	99.658	98.885	98.906	99.513	98.994	99.409	99.404	99.684	99.456	99.419
H	99.523	99.72	99.611	99.139	99.58	99.077	99.736	99.202	99.689	99.383	99.736	98.833

5505834338526080618064

	1	2	3	4	5	6	7	8	9	10	11	12
A	99.041	98.533	98.922	98.175	99.207	99.362	98.963	99.124	99.186	99.139	99.005	98.595
B	99.347	99.679	99.124	99.145	99.062	98.693	99.31	99.279	99.113	99.056	99.606	99.285
C	99.321	99.139	98.859	99.056	99.041	99.134	99.31	99.088	99.088	99.549	99.191	99.59
D	99.554	99.176	98.849	97.506	99.3	99.295	98.958	99.253	99.373	99.393	98.916	98.999
E	99.425	99.352	98.833	98.839	99.113	98.989	99.051	99.036	99.336	98.782	99.036	99.316
F	99.482	99.259	98.18	99.383	99.238	99.279	99.352	99.31	99.549	99.29	98.818	99.259
G	99.699	98.408	99.243	99.041	99.388	99.279	99.165	99.352	99.347	99.606	99.036	99.082
H	99.456	99.404	99.098	99.217	99.383	99.575	99.435	99.471	99.456	99.653	99.736	99.425

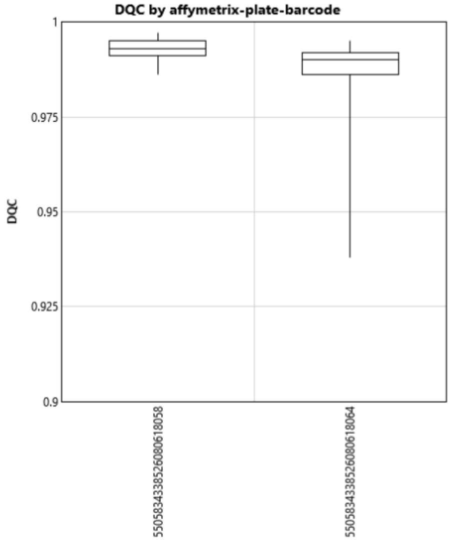

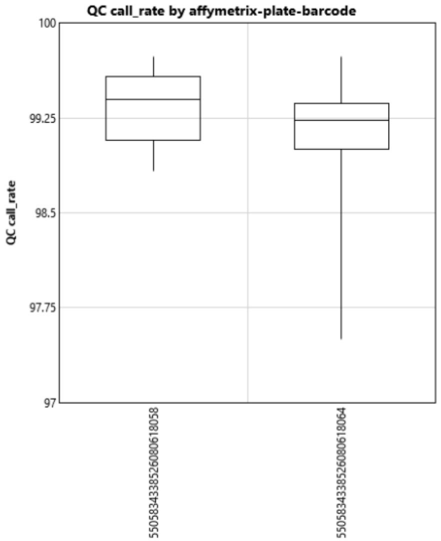

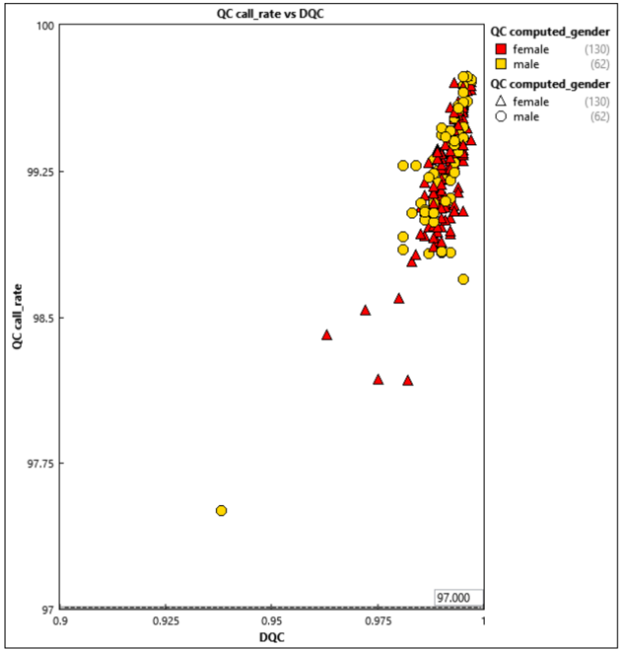

Supplement: Supplementary file 4 — Additional file 4: AxiomTM Analysis Suite final report. [file 12864_2021_8053_MOESM4_ESM.pdf]
